# Prescribed performance adaptive event-triggered consensus control for multiagent systems with input saturation

**DOI:** 10.3389/fnbot.2022.1103462

**Published:** 2023-01-19

**Authors:** Xia Yue, Jiarui Liu, Kairui Chen, Yuanqing Zhang, Zikai Hu

**Affiliations:** ^1^School of Mechanical and Electrical Engineering, Guangzhou University, Guangzhou, China; ^2^School of Computer and Information, Qiannan Normal University for Nationalities, Guizhou, China

**Keywords:** prescribed time, prescribed performance, event-triggered strategy, multiagent systems, input dead-zone, input saturation

## Abstract

In this paper, a prescribed performance adaptive event-triggered consensus control method is developed for a class of multiagent systems with the consideration of input dead zone and saturation. In practical engineering applications, systems are inevitably suffered from input saturation. In addition, input dead zone is widely existing. As the larger signal is limited and the smaller signal is difficult to effectively operate, system efficacious input encounters unknown magnitude limitations, which seriously impact system control performance and even lead to system instability. Furthermore, when constrained multiagent systems are required to converge quickly, the followers would achieve it with drastic and quick variation of states, which may violate the constraints and even cause security problems. To address those problems, an adaptive event-triggered consensus control is proposed. By constructing the transform function and the barrier Lyapunov function, while state constrained is guaranteed, multiagent systems quickly converge with prescribed performance. Finally, some examples are adopted to confirm the effectiveness of the proposed control method.

## 1. Introduction

With the rapid development of science and technology, multiagent systems are widely used in many fields, such as multirobot cooperation (Huang et al., 2018; Dai et al., 2021; Zhai et al., 2021), unmanned surface vehicles (Zhou et al., 2020; Gu et al., 2021; Huang et al., 2021), unmanned aerial vehicles (Gong et al., 2021; Tran et al., 2021; Zhou and Chen, 2021), and other fields. The multiagent system cooperative control mainly includes consensus, formation, and swarm, and the problem of consensus control, as the foundation of multiagent systems cooperative control, has received much attention from scholars (Cai et al., 2021; Ning et al., 2021; Su H. et al., 2021; Wang C. et al., 2021; Wei and Xiao, 2021). Cai et al. (2021) discussed the consensus problem of linear multiagent systems by constructing an adaptive coupling protocol. For second-order multiagent systems, a consensus algorithm was proposed based on sampled-data strategy by Su H. et al. (2021). Combined with the designed Nussbaum functions, the consensus problem of high-order nonlinear multiagent systems with unknown control directions was solved by an adaptive consensus tracking control scheme (Wang C. et al., 2021). It is noted that, while achieving systems consensus, taking the feature of convergence time into consideration is necessary.

Then, finite-time control was established. With faster convergence speed and robustness, it has received widespread attention (Li et al., 2020, 2021; Lu et al., 2021; Wang et al., 2022b). By finite-time control, it can be guaranteed that the systems converge within finite time. To achieve system convergence within predefined time for any initial states, the prescribed time control was extendedly proposed (Ren et al., 2020; Guo et al., 2021; Wang et al., 2021a; Chen et al., 2022; Gong et al., 2022). Focusing on first-order multiagent systems with single-integrator dynamics, a new control law with a scaling function was proposed (Chen et al., 2022). For the cluster lag consensus problem of multiagent systems, a distributed controller with time-varying gains was constructed by Ren et al. (2020). All of them achieved the aim that systems converge to preset states within predefined time, and the convergence time is independent of initial states. In addition, not only system convergence time but also transient performance and steady-state performance are important in practical engineering systems. It is a meaningful problem that how to develop a method to achieve multiagent system convergence with prescribed performance, especially while the system convergence time is settable.

Furthermore, many scholars have carried out a lot of research on nonlinear problems, such as input delay (Sun et al., 2022a), input hysteresis (Wang et al., 2021b), and time-varying mass (Sun et al., 2021). As the restriction of controller elements, input saturation is widely existing in systems. In prescribed time control, a large amount of energy is used to guarantee that the systems converge within a prescribed time. Especially when a short convergence time is prescribed, the input saturation problem becomes particularly acute. Therefore, input saturation is worth concerning when constructing a controller, many results were presented (Bai et al., 2020; Song et al., 2020; Cao et al., 2021; Min et al., 2021; Yang C. et al., 2021). Cao et al. (2021) used a smooth function and the mean-value theorem to approximate and transform the input saturation. Bai et al. (2020) constructed auxiliary systems to compensate for the influence of input saturation. On the contrary, due to the insensitivity of components to some small signals, input dead zone inevitably exists in systems. To reduce the impact of dead zone, some compensation methods were developed (Zhou et al., 2019; Ding et al., 2021; Lan et al., 2021; Jiang and Gao, 2022). For large-scale semi-Markovian jump interconnected systems with input dead zone, a local adaptive sliding mode control law was designed by Ding et al. (2021). For the output uncertainty problem caused by dead zone, a fuzzy algorithm was designed to deal with it by Lan et al. (2021). As the existence of input dead zone and saturation, many signals are difficult to perform effectively, control performance is seriously degraded, and it even affects system stability. To improve system reliability and practicability, it is significant to consider them when constructing control methods for multiagent systems.

With the improvement of safety performance requirements, the problem of state constraints has received more and more attention. Barrier Lyapunov functions, as a resultful way to constrain state, are used to solve various violation of constraints (Su W. et al., 2021; Zhao et al., 2021; Wang et al., 2022a; Wang N. et al., 2022). For a class of nonlinear systems, a new tracking control scheme was established based on barrier Lyapunov functions in Zhao et al. (2021). For high-order nonlinear multiagent systems, the barrier Lyapunov functions were used in the framework of the distributed adding-one-power-integrator control in Wang N. et al. (2022). Nevertheless, when systems required convergence in a short time, drastic variation of states is unavoidable, which may be against state constrained. How to balance state constraints and convergence time still needs to be further discussed.

Inspired by the above discussion, for constrained uncertain nonlinear multiagent systems with input dead zone and saturation, a prescribed time adaptive event-triggered consensus control with prescribed performance is developed. The main contributions are summarized as follows:

- To achieve the goal of system converging with prescribed performance within the prescribed time, the speed performance function is developed by incorporating the speed function into the performance function. By making blends and transformations, the transform function and the barrier Lyapunov function are constructed. Thus, prescribed time convergent processes of the systems satisfy prescribed performance, and the violation of state constrained is prevented.
- Both input dead zone and saturation exist in multiagent systems, which impact system control performance. As the model is non-smooth and the parameters are unknown, the design of the control method becomes difficult. Thus, the model is approximated by a non-affine smooth function and is further rewritten as the form of linear input and approximation error by the Mean Value Theorem. Moreover, an adaptive event-triggered control method is constructed to compensate them.
- To further improve the wide applicability and flexibility of the proposed method, the transform function and the barrier Lyapunov function are applied in each step, and all errors converge within the prescribed time.

The later sections are grouped as follows: Section 2 introduces the preliminary, Section 3 displays the control method design and stability analysis, Section 4 verifies the proposed control method through some simulation cases, and the conclusion is given in Section 5.

## 2. Preliminaries and problem description

### 2.1. System model

**Figure 1:**
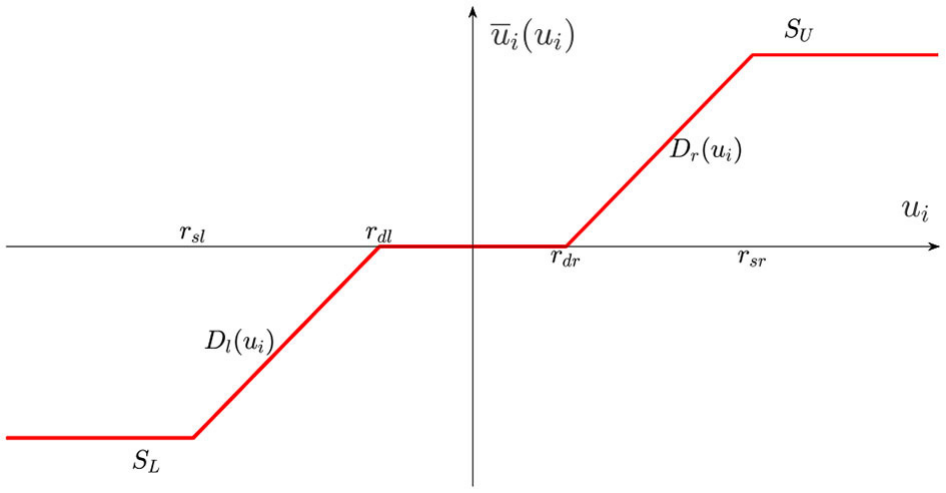
Input dead zone and saturation.

A class of multiagent systems included one virtual leader, and *M*(*M* > 2) followers are considered. The *i*-*th* (*i* = 1, 2, ⋯, *M*) follower is modeled as follows:


(1) $$\{\begin{array}{l} {\dot{x}}_{i,k}=x_{i,k+1}+f_{i,k}\left(X_{i,k}\right),\left(k=1,2,\cdots ,m-1\right)\\ {\dot{x}}_{i,m}={\bar{u}}_{i}\left(u_{i}\right)+f_{i,m}\left(X_{i,m}\right)\\ y_{i}=x_{i,1} \end{array}$$

where *x*_*i,k*_(*k* = 1, 2, ⋯, *m*) and *y*_*i*_ express the followers states and the output. *f*_*i,k*_(*X*_*i,k*_) represents the bounded external disturbances, and $X_{i,k}={\left[x_{i,1},x_{i,2},\cdots ,x_{i,k}\right]}^{T}\in R^{k}\left(k=1,2,\cdots ,m\right)$. In addition, *u*_*i*_ is input signal, ū_*i*_(*u*_*i*_) is system input with input dead zone and saturation as shown in Figure 1, and it can be described as follows:


(2) $${\bar{u}}_{i}\left(u_{i}\right)=\{\begin{array}{ll} S_{U}, & \;u_{i}>r_{sr}\\ D_{r}\left(u_{i}\right), & \;r_{dr}<u_{i}\leq r_{sr}\\ 0, & \;r_{dl}<u_{i}\leq r_{dr}\\ D_{l}\left(u_{i}\right), & \;r_{sl}<u_{i}\leq r_{dl}\\ S_{L}, & \;u_{i}\leq r_{sl} \end{array}$$

where *S*_*U*_ > 0 and *S*_*L*_ < 0 are system input saturation parameters, and *D*_*r*_(*u*_*i*_) and *D*_*l*_(*u*_*i*_) are unknown nonlinear functions. Then, and *r*_*sr*_ and *r*_*sl*_ are the saturation breakpoints, *r*_*dr*_ and *r*_*dl*_ are the dead-zone breakpoints. They satisfy *r*_*sl*_ < *r*_*dl*_ < 0 < *r*_*dr*_ < *r*_*sr*_.

Due to the nonlinear and non-smooth characteristics of ū_*i*_(*u*_*i*_), it is difficult to directly design control method. As a result, the ū_*i*_(*u*_*i*_) can be smoothly approximated as follows:


(3) $$\begin{array}{l} {ū}_{i}\left(u_{i}\right)=\kappa_{i}\left(u_{i}\right)+\varepsilon_{i}\left(u_{i}\right) \end{array}$$

where ε_*i*_(*u*_*i*_) = ū_*i*_(*u*_*i*_) − κ_*i*_(*u*_*i*_) expresses the approximation error, and it satisfies $|\varepsilon_{i}\left(u_{i}\right)|\leq {\bar{\varepsilon }}_{i}$. The smoothing function κ_*i*_(*u*_*i*_) can be defined as follows:


(4) $$\begin{array}{l} \begin{array}{c} \kappa_{i}\left(u_{i}\right)=\frac{S_{U}}{2}\tanh\theta_{1}\left(u_{i}-\frac{r_{dr}}{\theta_{1}}-\iota_{r}\right)-\frac{S_{L}}{2}\tanh\theta_{2}\left(u_{i}-\frac{r_{lr}}{\theta_{2}}+\iota_{l}\right)\\ \;+\frac{S_{U}}{2}\tanh\left(r_{dr}+\theta_{1}\iota_{r}\right)-\frac{S_{L}}{2}\tanh\left(r_{dl}-\iota_{l}\theta_{2}\right) \end{array} \end{array}$$

where ι_*r*_, ι_*l*_, θ_1_, and θ_2_ are positive parameters.

According to the Mean Value Theorem, the function $\kappa_{i}\left(u_{i}\right)={\dot{\kappa }}_{i}\left(u_{h}\right)\left(u_{i}-u_{i}^{0}\right)+\kappa_{i}\left(u_{i}^{0}\right)$ holds for $u_{h}=lu_{i}+\left(1-l\right)u_{i}^{0}$, where ${\dot{\kappa }}_{i}\left(u_{h}\right)$ is the derivative of smoothing function κ_*i*_(*u*_*h*_), and *l* is a constant satisfies 0 < *l* < 1. Let $u_{i}^{0}=0$, then κ_*i*_(*u*_*i*_) is described as $\kappa_{i}\left(u_{i}\right)={\dot{\kappa }}_{i}\left(u_{h}\right)u_{i}$. Then, define ${\dot{\kappa }}_{i}\left(u_{h}\right)=h_{i}$, and the system input ū_*i*_(*u*_*i*_) can be rewritten as follows:


(5) $$\begin{array}{l} {ū}_{i}\left(u_{i}\right)=h_{i}u_{i}+\varepsilon_{i}\left(u_{i}\right) \end{array}$$

### 2.2. Graph theory

The communication topology of multiagent system which includes *M*(*M* > 2) followers can be described as a digraph *G* = (*V, E*), and each follower can be expressed as a node. Then, *V* = {1, ⋯, *M*} denotes the node set, and *V*_*j*_ × *V*_*i*_ ∈ *E* represents the edge set between note *j* and note *i*. *A* = [_*a*_*ij*_]*M*×*M*_ represents the adjacency matrix among notes, where *a*_*ij*_ is the information transmission coefficient between note *j* and note *i*. If note *j* can get information from note *i*, then *a*_*ij*_ > 0. Otherwise, *a*_*ij*_ = 0. In addition, the Laplacian matrix *L* is defined as *L* = *D* − *A*, where the diagonal matrix is $D=diag\left[d_{1},d_{2},\cdots ,d_{M}\right]\in R^{M\times M}$ with $d_{i}=\sum_{j=1,j\neq i}^{M}a_{ij}$. For leader-follower multiagent systems, it can be represented by an augmented graph $Ḡ=\left(\bar{V},Ē\right)$, where $\bar{V}=\left\{0,1,\cdots ,M\right\}$ describes leader note 0 and followers note (1, ⋯, *M*), then ${\bar{V}}_{j}\times {\bar{V}}_{i}\in Ē$.

**Definition 1**. The definition of *i*-*th* follower's synchronization error can be expressed as follows:


(6) $$\begin{array}{l} z_{i,1}=\overset{M}{\underset{j=1}{\sum }}a_{ij}\left(y_{i}-y_{j}\right)+b_{i}\left(y_{i}-y_{0}\right) \end{array}$$

where *y*_0_ is the output signal of virtual leader, and *b*_*i*_ denotes the information transmission coefficient between virtual leader and follower *i*. If the follower *i* can receive information from the leader, *b*_*i*_ > 0. Otherwise, *b*_*i*_ = 0.

### 2.3. Radial basis function neural networks

For any continuous function $\varphi \left(\bar{X}\right)$ defined on a compact set Ω ∈ *R*^*p*^, it can be modeled by radial basis function neural networks (RBFNNs). Radial basis function neural networks are expressed as follows Sun et al. (2022b):


(7) $$\begin{array}{l} \varphi \left(\bar{X}\right)=K^{*T}\Phi \left(\bar{X}\right)+\tau \left(\bar{X}\right),|\tau \left(\bar{X}\right)|\leq \bar{\tau } \end{array}$$

where $\bar{X}={\left[x_{1},x_{2},\cdots ,x_{p}\right]}^{T}$ is input vector, and $\tau \left(\bar{X}\right)$ presents the approximation error, existing up bound $\bar{\tau }>0$ satisfying $|\tau \left(\bar{X}\right)|\leq \bar{\tau }$. Then, $\Phi \left(\bar{X}\right)={\left[\Phi_{1}\left(\bar{X}\right),\Phi_{2}\left(\bar{X}\right),\cdots ,\Phi_{q}\left(\bar{X}\right)\right]}^{T}$ expresses the basis function vector, and *q* > 1 is number of neural network node. $\Phi_{i}\left(\bar{X}\right)$ can be selected by the Gaussian functions as follows:


(8) $$\begin{array}{l} \Phi_{i}\left(\bar{X}\right)=\exp\left(-\frac{{\left||{\bar{X}}_{i}-X_{i}^{*}|\right|}^{2}}{\pi_{i}^{2}}\right),i=1,2,\cdots ,q \end{array}$$

where $X_{i}^{*}$ and π_*i*_ are the center and width of the Gaussian function.

K^*^ represents optimal weight value vector, and it can be described as follows:


(9) $$K^{*}=\arg\underset{K\in R^{p}}{\min}\left\{\underset{\bar{X}\in \Omega }{\sup}\left|\varphi \left(\bar{X}\right)-K^{T}\Phi \left(\bar{X}\right)\right|\right\}$$

where $K={\left[K_{1},K_{2},\cdots ,K_{q}\right]}^{T}$ is the weight vector.

### 2.4. Transform function

To constrain the system error within a prescribed region, the prescribed performance function is introduced as follows:


(10) $$\begin{array}{l} Z\left(t\right)=\left(Z^{0}-Z^{\infty }\right)\phi \left(t\right)+Z^{\infty } \end{array}$$

where *Z*^0^ > 0 and *Z*^∞^ > 0 present the initial value and the final value of the prescribed performance function. Then, ϕ(*t*) is a decay function, which satisfies ϕ(0) = 1 and $\underset{t\to \infty }{\lim}\phi \left(t\right)=0$. Prescribed performance of the system error *z*(*t*) satisfies |*z*(*t*)| ≤ *Z*(*t*).

To enhance system convergence rate and achieve the final prescribed performance within a prescribed time, the following speed function is introduced:


(11) $$\begin{array}{l} v\left(t\right)=\frac{{\left(T-t\right)}^{2}}{T^{2}},t\in \left(0,T\right] \end{array}$$

where *T* is a prescribed time that can be designed.

Combining Equations (10), (11), the speed performance function can be obtained as follows:


(12) $$\begin{array}{l} Z^{v}\left(t\right)=\left(Z^{0}-Z^{\infty }\right)\phi \left(t\right)v\left(t\right)+Z^{\infty } \end{array}$$

Noting that the inequality *Z*^*v*^(*t*) ≤ *Z*(*t*) is keeping holds. Existing parameters λ and *Q* satisfy λ ≤ *Z*^0^ and λ*Q*^−1^ ≤ *Z*^∞^, and such transform function is constructed as follows:


(13) $$g\left(t\right)=\{\begin{array}{ll} \frac{T^{2}\phi^{-1}\left(t\right)}{\left(1-Q^{-1}\right){\left(T-t\right)}^{2}+Q^{-1}T^{2}\phi^{-1}\left(t\right)}, & \;0\leq t<T\\ Q, & \;t\geq T \end{array}$$

**Remark 1**. According to the aforementioned deduction and analysis, it is known that the prescribed time *T* is independent of the system initial states and design parameters. Noting that λ*g*^−1^(*t*) ≤ *Z*^*v*^(*t*) is always holds, it is extremely significant for the later deduction and to prove.

### 2.5. Other preliminaries

**Assumption 1**. Zhang and Lewis (2012) the augmented graph Ḡ has a spanning tree, in which the root of the spanning tree is the virtual leader 0.

**Assumption 2**. The virtual leader output signal *y*_0_ is continuous function with up bound ȳ_0_ and has n-th order derivatives.

**Assumption 3**. Yang P. et al. (2021) define adjacency matrix as $B=diag\left[b_{1},b_{2},\cdots ,b_{M}\right]\in R^{M\times M}$. At least existing one *b*_*i*_ satisfies *b*_*i*_ > 0, so that *L* + *B* is nonsingular.

**Lemma 1**. Liu et al. (2018) for any λ ∈ *R* and *e* ∈ *R* satisfy |*e*| ≤ λ, the following inequality holds


(14) $$\begin{array}{l} \log\frac{\lambda^{2}}{\lambda^{2}-e^{2}}\leq \frac{e^{2}}{\lambda^{2}-e^{2}} \end{array}$$

**Lemma 2**. Wang et al. (2022c) for variables *m* ∈ *R* and *n* ∈ *R*, existing constants *a* > 0, *b* > 0, and *c* > 0 satisfy


(15) $$\begin{array}{l} |m{|}^{a}|n{|}^{b}\leq \frac{a}{a+b}c|m{|}^{a+b}+\frac{b}{a+b}c^{-\frac{a}{b}}|n{|}^{a+b} \end{array}$$

**Lemma 3**. Zhang and Lewis (2012) define $z_{M}={\left[z_{1,1},z_{2,1},\cdots ,z_{M,1}\right]}^{T}$, $Y_{M}={\left[y_{1},y_{2},\cdots ,y_{M}\right]}^{T}$, and $Y_{d}=\underset{M}{\underset{︸}{{\left[y_{d},y_{d},\cdots ,y_{d}\right]}^{T}}}$, satisfying


(16) $$\begin{array}{l} ||Y_{M}-Y_{d}||\leq \frac{\left||z_{M}|\right|}{\varsigma_{\min}} \end{array}$$

where ς_min_ is the minimum singular value of *L* + *B*, and *Y*_*M*_ − *Y*_*d*_ is track error.

## 3. Controller design and ability analysis

### 3.1. Prescribed performance adaptive event-triggered control design

Define the following coordinate transformations:


(17) $$\begin{array}{l} z_{i,k}=\;x_{i,k}-\alpha_{i,\left(k-1\right)},k=2,\cdots ,m \end{array}$$

(18) $$\begin{array}{l} e_{i,k}=\;g_{i}z_{i,k},k=1,2,\cdots ,m \end{array}$$

where *z*_*i*, 2_, *z*_*i*, 3_, ⋯, *z*_*i,m*_ represent error variables, and α_*i*, 1_, α_*i*, 2_, ⋯, α_*i,m*−1_ express virtual controllers.

**Step 1**: According to Equation (18), the derivative of *e*_*i*, 1_ can be obtained as follows:


(19) $$\begin{array}{l} \begin{array}{c} {ė}_{i,1}={ġ}_{i}z_{i,1}+g_{i}{ż}_{i,1}\\ \;={ġ}_{i}z_{i,1}+g_{i}\left(\left(b_{i}+d_{i}\right){ẏ}_{i}-b_{i}{ẏ}_{0}-\overset{M}{\underset{j=1}{\sum }}a_{ij}{ẏ}_{j}\right)\\ \;={ġ}_{i}z_{i,1}+g_{i}\left(\left(b_{i}+d_{i}\right)\left(z_{i,2}+\alpha_{i,1}+f_{i,1}\right)-b_{i}{ẏ}_{0}-\overset{M}{\underset{j=1}{\sum }}a_{ij}{ẏ}_{j}\right)\\ \;=g_{i}\left(\chi z_{i,1}+\left(b_{i}+d_{i}\right)\left(z_{i,2}+\alpha_{i,1}+f_{i,1}\right)-b_{i}{ẏ}_{0}-\overset{M}{\underset{j=1}{\sum }}a_{ij}{ẏ}_{j}\right) \end{array} \end{array}$$

where $\chi =g_{i}^{-1}{ġ}_{i}$

The following modified barrier Lyapunov function *V*_*i*, 1_ is constructed.


(20) $$\begin{array}{l} V_{i,1}=\frac{1}{2}\log\frac{\lambda_{i,1}^{2}}{\lambda_{i,1}^{2}-e_{i,1}^{2}}+\frac{1}{2\beta_{i,1}}{\tilde{\mu }}_{i,1}^{2} \end{array}$$

where λ_*i*, 1_ is the design parameter with $\lambda_{i,1}g_{i}^{-1}\left(t\right)\leq Z_{i,1}^{v}\left(t\right)$. It should be indicated that the inequality |*e*_*i*, 1_| ≤ λ_*i*, 1_ holds when *V*_*i*, 1_ is bounded. β_*i*, 1_ is a positive design parameter. ${\tilde{\mu }}_{i,1}=\mu_{i,1}-{\hat{\mu }}_{i,1}$ is a parameter estimation error, ${\hat{\mu }}_{i,1}$ is the estimation value of μ_*i*, 1_, and μ_*i*, 1_ will be defined later.

**Remark 2**. To multiagent system, prescribed time convergence progress meet prescribed performance while state constrained does not being violated, the transform function and barrier Lyapunov function are applied in Equations (18) and (20), and virtual controller and adaptive law will be developed to ensure *V*_*i*, 1_ is bounded. Moreover, similar application will be shown in each step to further enhance system performance.

Taking the time derivation of *V*_*i*, 1_ and combining with Equation (19), it can be obtained that


(21) $$\begin{array}{l} \begin{array}{c} {\dot{V}}_{i,1}=\frac{e_{i,1}g_{i}}{\lambda_{i,1}^{2}-e_{i,1}^{2}}\left(\chi z_{i,1}+\left(b_{i}+d_{i}\right)\left(z_{i,2}+\alpha_{i,1}+f_{i,1}\right)-b_{i}{ẏ}_{0}-\overset{M}{\underset{j=1}{\sum }}a_{ij}{ẏ}_{j}\right)\\ \;-\frac{1}{\beta_{i,1}}{\tilde{\mu }}_{i,1}{\dot{\hat{\mu }}}_{i,1}\\ \;=\frac{e_{i,1}g_{i}}{\lambda_{i,1}^{2}-e_{i,1}^{2}}\left(\left(b_{i}+d_{i}\right)\left(z_{i,2}+\alpha_{i,1}\right)+\varphi_{i,1}\left({\bar{X}}_{i,1}\right)\right)-\frac{1}{\beta_{i,1}}{\tilde{\mu }}_{i,1}{\dot{\hat{\mu }}}_{i,1} \end{array} \end{array}$$

where $\varphi_{i,1}\left({\bar{X}}_{i,1}\right)$ is smoothing function and is defined as follows:


(22) $$\begin{array}{l} \varphi_{i,1}\left({\bar{X}}_{i,1}\right)=\chi z_{i,1}+\left(b_{i}+d_{i}\right)f_{i,1}-b_{i}{ẏ}_{0}-\overset{N}{\underset{j=1}{\sum }}a_{ij}{ẏ}_{j} \end{array}$$

where ${\bar{X}}_{i,1}={\left[x_{i,1},x_{j,1},x_{j,2}\right]}^{T}$.

According to RBFNNs, it can be estimated with any given $\sigma_{i,1}\left({\bar{X}}_{i,1}\right)$


(23) $$\begin{array}{l} \varphi_{i,1}\left({\bar{X}}_{i,1}\right)=K_{i,1}^{*T}\Phi_{i,1}\left({\bar{X}}_{i,1}\right)+\tau_{i,1}\left({\bar{X}}_{i,1}\right),\tau_{i,1}\left({\bar{X}}_{i,1}\right)\leq {\bar{\tau }}_{i,1} \end{array}$$

**Remark 3**. The unknown and uncertainty of the multiagent system will bring difficulties to the design of the control method. Neural networks are introduced to approximate any unknown functions, which is handled by Young's inequality. In addition, selecting an optimal weight value vector K^*^ becomes difficult as the system gets more complex. Thus, the unknown parameter is estimated by an adaptive law ${\dot{\hat{\mu }}}_{i,1}$.

By utilizing **Lemma 2**, the following inequalities can be obtained:


(24) $$\begin{array}{l} \frac{|e_{i,1}g_{i}|\left(b_{i}+d_{i}\right)}{\lambda_{i,1}^{2}-e_{i,1}^{2}}z_{i,2}\leq \frac{{\left(b_{i}+d_{i}\right)}^{2}g_{i}^{2}e_{i,1}^{2}}{2{\left(\lambda_{i,1}^{2}-e_{i,1}^{2}\right)}^{2}}+\frac{z_{i,2}^{2}}{2} \end{array}$$

(25) $$\begin{array}{l} \frac{|e_{i,1}g_{i}|\varphi_{i,1}}{\lambda_{i,1}^{2}-e_{i,1}^{2}}\leq \frac{g_{i}^{2}e_{i,1}^{2}{\left||K_{i,1}^{*}|\right|}^{2}{\left||\Phi_{i,1}|\right|}^{2}}{2\ell_{i,1}^{2}{\left(\lambda_{i,1}^{2}-e_{i,1}^{2}\right)}^{2}}+\frac{\ell_{i,1}^{2}}{2}+\frac{g_{i}^{2}e_{i,1}^{2}}{2{\bar{\ell }}_{i,1}^{2}{\left(\lambda_{i,1}^{2}-e_{i,1}^{2}\right)}^{2}}+\frac{{\bar{\tau }}_{i,1}^{2}{\bar{\ell }}_{i,1}^{2}}{2} \end{array}$$

where ℓ_*i*,1_ > 0 and ${\bar{\ell }}_{i,1}>0$ are designed parameters.

Substituting into Equation (21), one has the following equation:


(26) $$\begin{array}{l} \begin{array}{c} {\dot{V}}_{i,1}\leq \frac{e_{i,1}g_{i}}{\lambda_{i,1}^{2}-e_{i,1}^{2}}\left(b_{i}+d_{i}\right)\alpha_{i,1}+\frac{g_{i}^{2}e_{i,1}^{2}{\left||K_{i,1}^{*}|\right|}^{2}{\left||\Phi_{i,1}|\right|}^{2}}{2\ell_{i,1}^{2}{\left(\lambda_{i,1}^{2}-e_{i,1}^{2}\right)}^{2}}+\frac{\ell_{i,1}^{2}}{2}\\ \;+\frac{g_{i}^{2}e_{i,1}^{2}}{2{\bar{\ell }}_{i,1}^{2}{\left(\lambda_{i,1}^{2}-e_{i,1}^{2}\right)}^{2}}\\ \;+\frac{{\bar{\tau }}_{i,1}^{2}{\bar{\ell }}_{i,1}^{2}}{2}+\frac{{\left(b_{i}+d_{i}\right)}^{2}g_{i}^{2}e_{i,1}^{2}}{2{\left(\lambda_{i,1}^{2}-e_{i,1}^{2}\right)}^{2}}+\frac{z_{i,2}^{2}}{2}-\frac{1}{\beta_{i,1}}{\tilde{\mu }}_{i,1}{\dot{\hat{\mu }}}_{i,1} \end{array} \end{array}$$

Define parameter $P_{i,1}=\frac{g_{i}^{2}}{2\left(\lambda_{i,1}^{2}-e_{i,1}^{2}\right)}\left(\frac{{\left||\Phi_{i,1}|\right|}^{2}}{\ell_{i,1}^{2}}+\frac{1}{{\bar{\ell }}_{i,1}^{2}}+{\left(b_{i}+d_{i}\right)}^{2}\right)$, the following inequality can be obtained:


(27) $$\begin{array}{l} \begin{array}{c} {\dot{V}}_{i,1}\leq \frac{e_{i,1}g_{i}}{\lambda_{i,1}^{2}-e_{i,1}^{2}}\left(\left(b_{i}+d_{i}\right)\alpha_{i,1}+z_{i,1}P_{i,1}\mu_{i,1}\right)+\frac{\ell_{i,1}^{2}}{2}+\frac{{\bar{\tau }}_{i,1}^{2}{\bar{\ell }}_{i,1}^{2}}{2}+\frac{z_{i,2}^{2}}{2}\\ \;-\frac{1}{\beta_{i,1}}{\tilde{\mu }}_{i,1}{\dot{\hat{\mu }}}_{i,1} \end{array} \end{array}$$

where $\mu_{i,1}=\max\left\{{\left||\overset{*}{\underset{i,1}{K}}|\right|}^{2},1\right\}$.

To guarantee the *V*_*i*,1_ bounded, constructing the virtual controller α_*i*,1_ and adaptive law ${\dot{\hat{\mu }}}_{i,1}$ as follows:


(28) $$\begin{array}{l} \alpha_{i,1}=\frac{1}{\left(b_{i}+d_{i}\right)}\left(-\gamma_{i,1}z_{i,1}-z_{i,1}{\hat{\mu }}_{i,1}P_{i,1}\right) \end{array}$$

(29) $$\begin{array}{l} {\dot{\hat{\mu }}}_{i,1}=\frac{e_{i,1}^{2}}{\lambda_{i,1}^{2}-e_{i,1}^{2}}\beta_{i,1}P_{i,1}-\delta_{i,1}{\hat{\mu }}_{i,1} \end{array}$$

where γ_*i*,1_ > 0 and δ_*i*,1_ > 0 are design parameters.

Substituting Equations (28), (29) into Equation (26), one has the following equation


(30) $$\begin{array}{l} {\dot{V}}_{i,1}\leq -\frac{\gamma_{i,1}e_{i,1}^{2}}{\lambda_{i,1}^{2}-e_{i,1}^{2}}+\frac{\delta_{i,1}}{\beta_{i,1}}{\tilde{\mu }}_{i,1}{\hat{\mu }}_{i,1}+\frac{\ell_{i,1}^{2}}{2}+\frac{{\bar{\tau }}_{i,1}^{2}{\bar{\ell }}_{i,1}^{2}}{2}+\frac{z_{i,2}^{2}}{2} \end{array}$$

**Step k (*k* = 2, 3, ⋯, *m* − 1)** : According to the definition of error *e*_*i,k*_ in Equation (18), the derivative of *e*_*i,k*_ can be obtained as follows:


(31) $$\begin{array}{l} \begin{array}{c} {ė}_{i,k}={ġ}_{i}z_{i,k}+g_{i}{ż}_{i,k}\\ \;={ġ}_{i}z_{i,k}+g_{i}\left(z_{i,k+1}+\alpha_{i,k}+f_{i,k}-{\dot{\alpha }}_{i,k-1}\right)\\ \;=g_{i}\left(\chi z_{i,k}+z_{i,k+1}+\alpha_{i,k}+f_{i,k}-{\dot{\alpha }}_{i,k-1}\right) \end{array} \end{array}$$

where ${\dot{\alpha }}_{i,k-1}=\overset{k-1}{\underset{j=1}{\sum }}\frac{\partial \alpha_{i,k-1}}{\partial x_{i,j}}{ẋ}_{i,j}+\overset{k-1}{\underset{j=1}{\sum }}\overset{M}{\underset{l=1}{\sum }}\frac{\partial \alpha_{i,k-1}}{\partial x_{l,j}}{ẋ}_{l,j}+\overset{k-1}{\underset{j=1}{\sum }}\frac{\partial \alpha_{i,k-1}}{\partial y_{0}}{ẏ}_{0}+\overset{k-1}{\underset{j=1}{\sum }}\frac{\partial \alpha_{i,k-1}}{\partial {\hat{\mu }}_{i,j}}{\dot{\hat{\mu }}}_{i,j}$.

The following barrier Lyapunov function *V*_*i,k*_ is constructed:


(32) $$\begin{array}{l} V_{i,k}=V_{i,k-1}+\frac{1}{2}\log\frac{\lambda_{i,k}^{2}}{\lambda_{i,k}^{2}-e_{i,k}^{2}}+\frac{1}{2\beta_{i,k}}{\tilde{\mu }}_{i,k}^{2} \end{array}$$

where λ_*i,k*_ is the design parameter with $\lambda_{i,k}g_{i}^{-1}\left(t\right)\leq Z_{i,k}^{v}\left(t\right)$, β_*i,k*_ is positive design parameter, ${\tilde{\mu }}_{i,k}=\mu_{i,k}-{\hat{\mu }}_{i,k}$ is parameter estimation error, ${\hat{\mu }}_{i,k}$ is the estimation value of μ_*i,k*_, and μ_*i,k*_ will be defined later.

Taking the time derivation of *V*_*i,k*_ and substituting Equation (31), the following equation can be obtained:


(33) $$\begin{array}{l} \begin{array}{c} {\dot{V}}_{i,k}={\dot{V}}_{i,k-1}+\frac{e_{i,k}g_{i}}{\lambda_{i,k}^{2}-e_{i,k}^{2}}\left(\chi z_{i,k}+z_{i,k+1}+\alpha_{i,k}+f_{i,k}-{\dot{\alpha }}_{i,k-1}\right)\\ \;-\frac{1}{\beta_{i,k}}{\tilde{\mu }}_{i,k}{\dot{\hat{\mu }}}_{i,k}\\ \;={\dot{V}}_{i,k-1}+\frac{e_{i,k}g_{i}}{\lambda_{i,k}^{2}-e_{i,k}^{2}}\left(z_{i,k+1}+\alpha_{i,k}+\varphi_{i,k}\left({\bar{X}}_{i,k}\right)\right)-\frac{1}{\beta_{i,k}}{\tilde{\mu }}_{i,k}{\dot{\hat{\mu }}}_{i,k} \end{array} \end{array}$$

where $\varphi_{i,k}\left({\bar{X}}_{i,k}\right)$ is smoothing function and is defined as follows:


(34) $$\begin{array}{l} \varphi_{i,k}\left({\bar{X}}_{i,k}\right)=\chi z_{i,k}+f_{i,k}-{\dot{\alpha }}_{i,k-1} \end{array}$$

where ${\bar{X}}_{i,k}={\left[x_{i,1},\ldots ,x_{i,k},x_{j,1},\ldots ,x_{j,k},{\hat{\mu }}_{i,1},\ldots ,{\hat{\mu }}_{i,k-1}\right]}^{T}$.

According to RBFNNs, the function can be estimated with any given $\tau_{i,k}\left({\bar{X}}_{i,k}\right)$


(35) $$\begin{array}{l} \varphi_{i,k}\left({\bar{X}}_{i,k}\right)=K_{i,k}^{*T}\Phi_{i,k}\left({\bar{X}}_{i,k}\right)+\tau_{i,k}\left({\bar{X}}_{i,k}\right),\tau_{i,k}\left({\bar{X}}_{i,k}\right)\leq {\bar{\tau }}_{i,k} \end{array}$$

By utilizing **Lemma 2**, the following inequalities can be deduced:


(36) $$\begin{array}{l} \frac{|e_{i,k}g_{i}|z_{i,k+1}}{\lambda_{i,k}^{2}-e_{i,k}^{2}}\leq \frac{g_{i}^{2}e_{i,k}^{2}}{2{\left(\lambda_{i,k}^{2}-e_{i,k}^{2}\right)}^{2}}+\frac{z_{i,k+1}^{2}}{2} \end{array}$$

(37) $$\begin{array}{l} \frac{|e_{i,k}g_{i}|\varphi_{i,k}}{\lambda_{i,k}^{2}-e_{i,k}^{2}}\leq \frac{g_{i}^{2}e_{i,k}^{2}{\left||K_{i,k}^{*}|\right|}^{2}{\left||\Phi_{i,k}|\right|}^{2}}{2\ell_{i,k}^{2}{\left(\lambda_{i,k}^{2}-e_{i,k}^{2}\right)}^{2}}+\frac{\ell_{i,k}^{2}}{2}+\frac{g_{i}^{2}e_{i,k}^{2}}{2{\bar{\ell }}_{i,k}^{2}{\left(\lambda_{i,k}^{2}-e_{i,k}^{2}\right)}^{2}}+\frac{{\bar{\tau }}_{i,k}^{2}{\bar{\ell }}_{i,k}^{2}}{2} \end{array}$$

where ℓ_*i,k*_ > 0 and ${\bar{\ell }}_{i,k}>0$ are designed parameters.

Substituting the inequalities into Equation (33), hence


(38) $$\begin{array}{l} \begin{array}{c} {\dot{V}}_{i,k}\leq {\dot{V}}_{i,k-1}+\frac{e_{i,k}g_{i}}{\lambda_{i,k}^{2}-e_{i,k}^{2}}\alpha_{i,k}+\frac{g_{i}^{2}e_{i,k}^{2}{\left||K_{i,k}^{*}|\right|}^{2}{\left||\Phi_{i,k}|\right|}^{2}}{2\ell_{i,k}^{2}{\left(\lambda_{i,k}^{2}-e_{i,k}^{2}\right)}^{2}}\\ \;+\frac{\ell_{i,k}^{2}}{2}+\frac{g_{i}^{2}e_{i,k}^{2}}{2{\bar{\ell }}_{i,k}^{2}{\left(\lambda_{i,k}^{2}-e_{i,k}^{2}\right)}^{2}}\\ \;+\frac{{\bar{\tau }}_{i,k}^{2}{\bar{\ell }}_{i,k}^{2}}{2}+\frac{g_{i}^{2}e_{i,k}^{2}}{2{\left(\lambda_{i,k}^{2}-e_{i,k}^{2}\right)}^{2}}+\frac{z_{i,k+1}^{2}}{2}-\frac{1}{\beta_{i,k}}{\tilde{\mu }}_{i,k}{\dot{\hat{\mu }}}_{i,k} \end{array} \end{array}$$

Define $P_{i,k}=\frac{e_{i,k}g_{i,k}}{2z_{i,k}\left(\lambda_{i,k}^{2}-e_{i,k}^{2}\right)}\left(\frac{{\left||\Phi_{i,k}|\right|}^{2}}{\ell_{i,k}^{2}}+\frac{1}{{\bar{\ell }}_{i,k}^{2}}+1\right)$ and $\mu_{i,k}=\max\left\{{\left||\overset{*}{\underset{i,k}{K}}|\right|}^{2},1\right\}$, one has


(39) $$\begin{array}{l} \begin{array}{c} {\dot{V}}_{i,k}\leq \frac{e_{i,k}g_{i}}{\lambda_{i,k}^{2}-e_{i,k}^{2}}\left(\alpha_{i,k}+z_{i,k}P_{i,k}\mu_{i,k}\right)+\frac{\ell_{i,k}^{2}}{2}+\frac{{\bar{\tau }}_{i,k}^{2}{\bar{\ell }}_{i,k}^{2}}{2}+\frac{z_{i,k+1}^{2}}{2}\\ \;-\frac{1}{\beta_{i,k}}{\tilde{\mu }}_{i,k}{\dot{\hat{\mu }}}_{i,k} \end{array} \end{array}$$

To guarantee the *V*_*i,k*_ bounded, constructing the virtual controller α_*i,k*_ and adaptive law ${\dot{\hat{\mu }}}_{i,k}$ as follows:


(40) $$\begin{array}{l} \alpha_{i,k}=-\gamma_{i,k}z_{i,k}-z_{i,k}{\hat{\mu }}_{i,k}P_{i,k}-\frac{\lambda_{i,k}^{2}-e_{i,k}^{2}}{g_{i}^{2}}z_{i,k} \end{array}$$

(41) $$\begin{array}{l} {\dot{\hat{\mu }}}_{i,k}=\frac{e_{i,k}^{2}}{\lambda_{i,k}^{2}-e_{i,k}^{2}}\beta_{i,k}P_{i,k}-\delta_{i,k}{\hat{\mu }}_{i,k} \end{array}$$

where γ_*i,k*_ > 0 and δ_*i,k*_ > 0 are design parameters.

Substituting Equations (40), (41) into Equation (39), it can be obtained as follows:


(42) $$\begin{array}{l} \begin{array}{c} {\dot{V}}_{i,k}\leq {\dot{V}}_{i,k-1}-\frac{\gamma_{i,k}e_{i,k}^{2}}{\lambda_{i,k}^{2}-e_{i,k}^{2}}+\frac{\delta_{i,k}}{\beta_{i,k}}{\tilde{\mu }}_{i,k}{\hat{\mu }}_{i,k}+\frac{\ell_{i,k}^{2}}{2}+\frac{{\bar{\tau }}_{i,k}^{2}{\bar{\ell }}_{i,k}^{2}}{2}+\frac{z_{i,k+1}^{2}}{2}-\frac{z_{i,k}^{2}}{2}\\ =-\overset{k}{\underset{j=1}{\sum }}\frac{\gamma_{i,j}e_{i,j}^{2}}{\lambda_{i,j}^{2}-e_{i,j}^{2}}+\overset{k}{\underset{j=1}{\sum }}\frac{\delta_{i,j}}{\beta_{i,j}}{\tilde{\mu }}_{i,j}{\hat{\mu }}_{i,j}+\overset{k}{\underset{j=1}{\sum }}\left(\frac{\ell_{i,j}^{2}}{2}+\frac{{\bar{\tau }}_{i,j}^{2}{\bar{\ell }}_{i,j}^{2}}{2}\right)+\frac{z_{i,k+1}^{2}}{2} \end{array} \end{array}$$

**Step m**: To relieve system communication pressure, an adaptive event-triggered strategy is constructed as follows:


(43) $$\{\begin{array}{c} \varpi_{i}\left(t\right)=-\left(1+\zeta_{i}\right)\left({\bar{\alpha }}_{i}\tanh\left(\frac{z_{i,m}{\bar{\alpha }}_{i}}{o_{i}}\right)+\frac{z_{i,m}}{2{\left(1-\zeta_{i}\right)}^{2}}\right)\\ u_{i}\left(t\right)=\varpi_{i}\left(t_{\omega }\right),\forall t\in \left[t_{\omega },t_{\omega +1}\right)\\ \Delta_{i}\left(t\right)=\varpi_{i}\left(t\right)-u_{i}\left(t\right)\\ t_{\omega +1}=\inf\left\{t\in R|\left|\Delta_{i}\left(t\right)\right|\geq \zeta_{i}\left|{\bar{u}}_{i}\left(t\right)\right|+\sigma_{i}\right\} \end{array}$$

where 0 < ζ_*i*_ < 1, σ_*i*_ > 0, and *o*_*i*_ > 0 are design parameters. The intermediate signal ${\bar{\alpha }}_{i}$ is defined as follows:


(44) $$\begin{array}{l} {\bar{\alpha }}_{i}=\eta_{i}\alpha_{i,m} \end{array}$$

where $\eta_{i}=h_{i}^{-1}$, and α_*i,m*_ is virtual controller, which will be constructed later. Consider *h*_*i*_ is unknown constant, by utilizing ${\hat{\eta }}_{i,m}$ to estimate η_*i,m*_, and ${\tilde{\eta }}_{i,m}=\eta_{i,m}-{\hat{\eta }}_{i,m}$ is the estimation error. Then, ${\bar{\alpha }}_{i}$ can be rewritten as follows:


(45) $$\begin{array}{l} {\bar{\alpha }}_{i}={\hat{\eta }}_{i}\alpha_{i,m} \end{array}$$

**Remark 4**. Consider the restricted communication resources, an event-triggered scheme (Equation 43) is established to reduce unnecessary communication transmission. At the same time, an intermediate signal with adaptive law is established in Equation (44) to compensate the impact of dead zone and saturation.

According to Equation (43), one has


(46) $$\begin{array}{l} u_{i}=\frac{{\bar{\omega }}_{i}-v_{i,1}\sigma_{i}}{1+v_{i,2}\zeta_{i}} \end{array}$$

where |*v*_*i*,1_| ≤ 1, |*v*_*i*,2_| ≤ 1.

For any constant *x* ∈ *R* and *y* > 0, the inequality $0\leq |x|-x\tanh\left(\frac{x}{y}\right)\leq 0.2875y$ holds. Then, it can be obtained as follows:


(47) $$\begin{array}{l} z_{i,m}\left(u_{i}\right)=-z_{i,m}(\frac{\left(1+\zeta_{i}\right){\bar{\alpha }}_{i}}{1+v_{i,2}\zeta_{i}}\tanh\left(\frac{z_{i,m}{\bar{\alpha }}_{i}}{o_{i}}\right)\\ \;+\frac{\left(1+\zeta_{i}\right)z_{i,m}}{2\left(1+v_{i,2}\zeta_{i}\right){\left(1-\zeta_{i}\right)}^{2}}+\frac{v_{i,2}\sigma_{i}}{1+v_{i,2}\zeta_{i}})\\ \;\leq \left|z_{i,m}{\bar{\alpha }}_{i}\right|-\left|z_{i,m}{\bar{\alpha }}_{i}\right|-z_{i,m}{\bar{\alpha }}_{i}\tanh\left(\frac{z_{i,m}{\bar{\alpha }}_{i}}{o_{i}}\right)-\frac{e_{i,m}^{2}}{2{\left(1-\zeta_{i}\right)}^{2}}\\ \;+\left|\frac{z_{i,m}\sigma_{i}}{\left(1-\zeta_{i}\right)}\right|\\ \;\leq z_{i,m}{\bar{\alpha }}_{i}+0.2785o_{i}+\frac{\sigma_{i}^{2}}{2} \end{array}$$

According to Equation (18), the derivative of *e*_*i,m*_ can be obtained as follows:


(48) $$\begin{array}{l} \begin{array}{c} {ė}_{i,m}={ġ}_{i}z_{i,m}+g_{i}{ż}_{i,m}\\ \;={ġ}_{i}z_{i,m}+g_{i}\left({ū}_{i}+f_{i,m}-{\dot{\alpha }}_{i,m-1}\right)\\ \;=g_{i}\left(\chi z_{i,m}+h_{i}u_{i}+\varepsilon_{i}+f_{i,m}-{\dot{\alpha }}_{i,m-1}\right) \end{array} \end{array}$$

where ${\dot{\alpha }}_{i,m-1}=\overset{m-1}{\underset{j=1}{\sum }}\frac{\partial \alpha_{i,m-1}}{\partial x_{i,j}}{ẋ}_{i,j}+\overset{m-1}{\underset{j=1}{\sum }}\overset{M}{\underset{l=1}{\sum }}\frac{\partial \alpha_{i,m-1}}{\partial x_{l,j}}{ẋ}_{l,j}+\overset{m-1}{\underset{j=1}{\sum }}\frac{\partial \alpha_{i,m-1}}{\partial y_{0}}{ẏ}_{0}+\overset{m-1}{\underset{j=1}{\sum }}\frac{\partial \alpha_{i,m-1}}{\partial {\hat{\mu }}_{i,j}}{\dot{\hat{\mu }}}_{i,j}$.

The following barrier Lyapunov function *V*_*i,m*_ is constructed:


(49) $$\begin{array}{l} V_{i,m}=V_{i,m-1}+\frac{1}{2}\log\frac{\lambda_{i,m}^{2}}{\lambda_{i,m}^{2}-e_{i,m}^{2}}+\frac{1}{2\beta_{i,m}}{\tilde{\mu }}_{i,m}^{2}+\frac{h_{i}}{2\rho_{i,m}}{\tilde{\eta }}_{i,m}^{2} \end{array}$$

where λ_*i,m*_ is the design parameter with $\lambda_{i,m}g^{-1}\left(t\right)\leq Z_{i,m}^{v}\left(t\right)$, β_*i,m*_ and ρ_*i,m*_ are positive design and positive parameter. ${\tilde{\mu }}_{i,m}=\mu_{i,m}-{\hat{\mu }}_{i,m}$ is parameter estimation error, ${\hat{\mu }}_{i,m}$ is the estimation value of μ_*i,m*_, and μ_*i,m*_ will be defined later.

Taking the time derivation of *V*_*i,m*_, it can be obtained as follows:


(50) $$\begin{array}{l} \begin{array}{c} {\dot{V}}_{i,m}={\dot{V}}_{i,m-1}+\frac{e_{i,m}g_{i}}{\lambda_{i,m}^{2}-e_{i,m}^{2}}\left(\chi z_{i,m}+h_{i}u_{i}+\varepsilon_{i}+f_{i,m}-{\dot{\alpha }}_{i,m-1}\right)\\ \;-\frac{1}{\beta_{i,m}}{\tilde{\mu }}_{i,m}{\dot{\hat{\mu }}}_{i,m}-\frac{h_{i}}{\rho_{i,m}}{\tilde{\eta }}_{i,m}{\dot{\hat{\eta }}}_{i,m} \end{array} \end{array}$$

Combined with Equation (47), one has


(51) $$\begin{array}{l} \begin{array}{c} {\dot{V}}_{i,m}={\dot{V}}_{i,m-1}+\frac{e_{i,m}g_{i}}{\lambda_{i,m}^{2}-e_{i,m}^{2}}(\chi z_{i,m}+\alpha_{i,m}-h_{i}{\hat{\eta }}_{i}\alpha_{i,m}+\frac{0.2785h_{i}o_{i}}{e_{i,m}}\\ \;+\frac{h_{i}\sigma_{i}^{2}}{2e_{i,m}}\\ \;+\varepsilon_{i}+f_{i,m}-{\dot{\alpha }}_{i,m-1})-\frac{1}{\beta_{i,m}}{\tilde{\mu }}_{i,m}{\dot{\hat{\mu }}}_{i,m}-\frac{h_{i}}{\rho_{i,m}}{\tilde{\eta }}_{i,m}{\dot{\hat{\eta }}}_{i,m} \end{array} \end{array}$$

Define the smoothing function $\varphi_{i,m}\left({\bar{X}}_{i,m}\right)$ as follows:


(52) $$\begin{array}{l} \begin{array}{c} \varphi_{i,m}\left({\bar{X}}_{i,m}\right)=\chi z_{i,m}+\frac{0.2785h_{i}o_{i}}{e_{i,m}}+\frac{h_{i}\sigma_{i}^{2}}{2e_{i,m}}+f_{i,m}-{\dot{\alpha }}_{i,m-1}\\ \;+\frac{{\bar{\varepsilon }}_{i}^{2}\left(\lambda_{i,m}^{2}-e_{i,m}^{2}\right)}{2e_{i,m}g_{i}} \end{array} \end{array}$$

where ${\dot{\alpha }}_{i,m-1}=\sum_{j=1}^{m-1}\frac{\partial \alpha_{i,m-1}}{\partial x_{i,j}}{ẋ}_{i,j}+\sum_{j=1}^{m-1}\sum_{l=1}^{M}\frac{\partial \alpha_{i,m-1}}{\partial x_{l,j}}{ẋ}_{l,j}+\sum_{j=1}^{m-1}\frac{\partial \alpha_{i,m-1}}{\partial y_{0}}{ẏ}_{0}+\sum_{j=1}^{m-1}\frac{\partial \alpha_{i,m-1}}{\partial {\hat{\mu }}_{i,j}}{\dot{\hat{\mu }}}_{i,j}$.

By utilizing **Lemma 2**, the following inequalities can be deduced as follows:


(53) $$\begin{array}{l} \frac{e_{i,m}g_{i}}{\lambda_{i,m}^{2}-e_{i,m}^{2}}\varepsilon_{i}\leq \frac{e_{i,m}^{2}g_{i}^{2}}{2{\left(\lambda_{i,m}^{2}-e_{i,m}^{2}\right)}^{2}}+\frac{{\bar{\varepsilon }}_{i}^{2}}{2} \end{array}$$

According to the RBFNNs, the function can be estimated with any given $\tau_{i,m}\left({\bar{X}}_{i,m}\right)$


(54) $$\begin{array}{l} \varphi_{i,m}\left({\bar{X}}_{i,m}\right)=K_{i,m}^{*T}\Phi_{i,m}\left({\bar{X}}_{i,m}\right)+\tau_{i,m}\left({\bar{X}}_{i,m}\right),\tau_{i,m}\left({\bar{X}}_{i,m}\right)\leq {\bar{\tau }}_{i,m} \end{array}$$

where ${\bar{X}}_{i,m}={\left[x_{i,1},\ldots ,x_{i,m},x_{j,1},\ldots ,x_{j,m},{\hat{\mu }}_{i,1},\ldots ,{\hat{\mu }}_{i,m-1}\right]}^{T}$.

According to **Lemma 2**, the following inequalities can be obtained:


(55) $$\begin{array}{l} \begin{array}{c} \frac{|e_{i,m}g_{i}|\varphi_{i,m}}{\lambda_{i,m}^{2}-e_{i,m}^{2}}\leq \frac{g_{i}^{2}e_{i,m}^{2}{\left||K_{i,m}^{*}|\right|}^{2}{\left||\Phi_{i,m}|\right|}^{2}}{2\ell_{i,m}^{2}{\left(\lambda_{i,m}^{2}-e_{i,m}^{2}\right)}^{2}}+\frac{\ell_{i,m}^{2}}{2}+\frac{g_{i}^{2}e_{i,m}^{2}}{2{\bar{\ell }}_{i,m}^{2}{\left(\lambda_{i,m}^{2}-e_{i,m}^{2}\right)}^{2}}\\ \;+\frac{{\bar{\tau }}_{i,m}^{2}{\bar{\ell }}_{i,m}^{2}}{2} \end{array} \end{array}$$

where ℓ_*i,m*_ > 0 and ${\bar{\ell }}_{i,m}>0$ are designed parameters.

Substituting the inequalities into Equation (51), one has


(56) $$\begin{array}{l} \begin{array}{c} {\dot{V}}_{i,m}\leq {\dot{V}}_{i,m-1}+\frac{e_{i,m}g_{i}}{\lambda_{i,m}^{2}-e_{i,m}^{2}}\alpha_{i,m}+\frac{g_{i}^{2}e_{i,m}^{2}{\left||K_{i,m}^{*}|\right|}^{2}{\left||\Phi_{i,m}|\right|}^{2}}{2\ell_{i,m}^{2}{\left(\lambda_{i,m}^{2}-e_{i,m}^{2}\right)}^{2}}+\frac{\ell_{i,m}^{2}}{2}\\ \;+\frac{g_{i}^{2}e_{i,m}^{2}}{2{\bar{\ell }}_{i,m}^{2}{\left(\lambda_{i,m}^{2}-e_{i,m}^{2}\right)}^{2}}+\frac{{\bar{\tau }}_{i,m}^{2}{\bar{\ell }}_{i,m}^{2}}{2}\\ \;+\frac{e_{i,m}^{2}g_{i}^{2}}{2{\left(\lambda_{i,m}^{2}-e_{i,m}^{2}\right)}^{2}}-\frac{1}{\beta_{i,m}}{\tilde{\mu }}_{i,m}{\dot{\hat{\mu }}}_{i,m}\\ \;-h_{i}{\tilde{\eta }}_{i,m}\left(\frac{e_{i,m}g_{i}}{\lambda_{i,m}^{2}-e_{i,m}^{2}}\alpha_{i,m}+\rho_{i,m}^{-1}{\dot{\hat{\eta }}}_{i,m}\right) \end{array} \end{array}$$

Define $P_{i,m}=\frac{g_{i}^{2}}{2\left(\lambda_{i,m}^{2}-e_{i,m}^{2}\right)}\left(\frac{{\left||\Phi_{i,m}|\right|}^{2}}{\ell_{i,m}^{2}}+\frac{1}{{\bar{\ell }}_{i,m}^{2}}+1\right)$ and $\mu_{i,m}=\max\left\{{\left||\overset{*}{\underset{i,m}{K}}|\right|}^{2},1\right\}$, it can be deduced as follows:


(57) $$\begin{array}{l} \begin{array}{c} {\dot{V}}_{i,m}\leq {\dot{V}}_{i,m-1}+\frac{e_{i,m}g_{i}}{\lambda_{i,m}^{2}-e_{i,m}^{2}}\left(\alpha_{i,m}+z_{i,m}P_{i,m}\mu_{i,m}\right)+\frac{\ell_{i,m}^{2}}{2}\\ \;+\frac{{\bar{\tau }}_{i,m}^{2}{\bar{\ell }}_{i,m}^{2}}{2}-\frac{1}{\beta_{i,m}}{\tilde{\mu }}_{i,m}{\dot{\hat{\mu }}}_{i,m}\\ \;-h_{i}{\tilde{\eta }}_{i,m}\left(\frac{e_{i,m}g_{i}}{\lambda_{i,m}^{2}-e_{i,m}^{2}}\alpha_{i,m}+\rho_{i,m}^{-1}{\dot{\hat{\eta }}}_{i,m}\right) \end{array} \end{array}$$

To guarantee the *V*_*i,m*_ bounded, constructing the virtual controller α_*i,m*_ and adaptive laws ${\dot{\hat{\mu }}}_{i,m}$ and ${\dot{\hat{\eta }}}_{i,m}$ as follows:


(58) $$\begin{array}{l} \alpha_{i,m}=-\gamma_{i,m}z_{i,m}-z_{i,m}{\hat{\mu }}_{i,m}P_{i,m}-\frac{\lambda_{i,m}^{2}-e_{i,m}^{2}}{g_{i}^{2}}z_{i,m} \end{array}$$

(59) $$\begin{array}{l} {\dot{\hat{\mu }}}_{i,m}=\frac{e_{i,m}^{2}}{\lambda_{i,m}^{2}-e_{i,m}^{2}}\beta_{i,m}P_{i,m}-\delta_{i,m}{\hat{\mu }}_{i,m} \end{array}$$

(60) $$\begin{array}{l} {\dot{\hat{\eta }}}_{i,m}=-\frac{e_{i,m}g_{i}\rho_{i,m}}{\lambda_{i,m}^{2}-e_{i,m}^{2}}\alpha_{i,m}-\xi_{i,m}{\hat{\eta }}_{i,m} \end{array}$$

where γ_*i,m*_ > 0, δ_*i,m*_ > 0, and ξ_*i,m*_ > 0.

**Remark 5**. Because the input dead-zone and saturation nonlinearities are unknown, parameters of them are difficult to obtained. Thus, an intermediate signal ${\bar{\alpha }}_{i}$ with adaptive law is designed to compensate them, and the approximation error is simultaneously handled by RBFNNs and adaptive law. The design difficulties caused by unknown parameters are effectively avoided.

Substituting Equations (58)–(60) produces Equation (57):


(61) $$\begin{array}{l} \begin{array}{c} {\dot{V}}_{i,m}\leq {\dot{V}}_{i,m-1}-\frac{\gamma_{i,m}e_{i,m}^{2}}{\lambda_{i,m}^{2}-e_{i,m}^{2}}+\frac{\delta_{i,m}}{\beta_{i,m}}{\tilde{\mu }}_{i,m}{\hat{\mu }}_{i,m}\\ \;+\frac{\xi_{i,m}h_{i}}{\rho_{i,m}}{\tilde{\eta }}_{i,m}{\hat{\eta }}_{i,m}+\frac{\ell_{i,m}^{2}}{2}+\frac{{\bar{\tau }}_{i,m}^{2}{\bar{\ell }}_{i,m}^{2}}{2}-\frac{z_{i,m}^{2}}{2}\\ \;=-\overset{m}{\underset{j=1}{\sum }}\frac{\gamma_{i,j}e_{i,j}^{2}}{\lambda_{i,j}^{2}-e_{i,j}^{2}}+\overset{m}{\underset{j=1}{\sum }}\frac{\delta_{i,j}}{\beta_{i,j}}{\tilde{\mu }}_{i,j}{\hat{\mu }}_{i,j}+\frac{\xi_{i,m}h_{i}}{\rho_{i,m}}{\tilde{\eta }}_{i,m}{\hat{\eta }}_{i,m}\\ \;+\overset{m}{\underset{j=1}{\sum }}\left(\frac{\ell_{i,j}^{2}}{2}+\frac{{\bar{\tau }}_{i,j}^{2}{\bar{\ell }}_{i,j}^{2}}{2}\right) \end{array} \end{array}$$

According to **Lemma 1** and **Lemma 2**, the following inequalities hold:


(62) $$\begin{array}{l} \log\frac{\lambda_{i,j}^{2}}{\lambda_{i,j}^{2}-e_{i,j}^{2}}\leq \frac{e_{i,j}^{2}}{\lambda_{i,j}^{2}-e_{i,j}^{2}} \end{array}$$

(63) $$\begin{array}{l} {\tilde{\eta }}_{i,m}{\hat{\eta }}_{i,m}\leq \frac{1}{2}\eta_{i,m}^{2}-\frac{1}{2}{\hat{\eta }}_{i,m}^{2} \end{array}$$

(64) $$\begin{array}{l} {\tilde{\mu }}_{i,j}{\hat{\mu }}_{i,j}\leq \frac{1}{2}\mu_{i,j}^{2}-\frac{1}{2}{\hat{\mu }}_{i,j}^{2} \end{array}$$

Based on Equations (61)–(64) can be converted as follows:


(65) $$\begin{array}{l} \begin{array}{c} {\dot{V}}_{i,m}\leq -\overset{m}{\underset{j=1}{\sum }}\gamma_{i,j}\log\frac{\lambda_{i,j}^{2}}{\lambda_{i,j}^{2}-e_{i,j}^{2}}-\overset{m}{\underset{j=1}{\sum }}\frac{\delta_{i,j}}{2\beta_{i,j}}\mu_{i,j}^{2}-\frac{\xi_{i,m}h_{i}}{2\rho_{i,m}}\eta_{i,m}^{2}\\ \;+\overset{m}{\underset{j=1}{\sum }}\left(\frac{\delta_{i,j}}{2\beta_{i,j}}{\hat{\mu }}_{i,j}^{2}+\frac{\ell_{i,j}^{2}}{2}+\frac{{\bar{\tau }}_{i,j}^{2}{\bar{\ell }}_{i,j}^{2}}{2}\right)+\frac{\xi_{i,m}h_{i}}{2\rho_{i,m}}{\hat{\eta }}_{i,m}^{2}\\ \;\leq -\Gamma_{i}V_{i,m}+\Upsilon_{i} \end{array} \end{array}$$

where $\Upsilon_{i}=\overset{m}{\underset{j=1}{\sum }}\left(\frac{\delta_{i,j}}{2\beta_{i,j}}{\hat{\mu }}_{i,j}^{2}+\frac{\ell_{i,j}^{2}}{2}+\frac{{\bar{\tau }}_{i,j}^{2}{\bar{\ell }}_{i,j}^{2}}{2}\right)+\frac{\xi_{i,m}h_{i}}{2\rho_{i,m}}{\hat{\eta }}_{i,m}^{2}$, $\Gamma_{i}=\min\left\{\frac{\gamma_{i,j}}{2},\delta_{i,j},\xi_{i,m},j=1,2,\cdots ,m\right\}$.

### 3.2. System stability analysis

**Theorem 1**. For uncertain nonlinear multiagent systems with input dead zone and saturation, by constructed virtual controllers (Equations 28, 40, and 58), adaptive laws (Equation 29, 41, 59, and 60), and event-triggered strategy (Equation 43), the following results can be achieved:

(1) All the signals of systems are bounded, and system error *z*_*i,j*_ converges to prescribed regions $\left\{z_{i,j}||z_{i,j}|\leq Q^{-1}\lambda_{i,j}\right\}$ within prescribed time *T*.
(2) System performance satisfies prescribed performance function *Z*_*i,j*_(*t*) and system states are fulfilling the constraints.
(3) There exits the minimum interval time between any twice triggering, so Zeno Behavior can be surely avoided.

*Proof of Theorem 1 (1)*. The following total Lyapunov function is constructed:


(66) $$\begin{array}{l} V=\overset{M}{\underset{i=1}{\sum }}V_{i,m} \end{array}$$

According to Equation (65), the following equation is obtained:


(67) $$\begin{array}{l} \dot{V}\leq -\Gamma V+\Upsilon \end{array}$$

where $\Upsilon =\overset{M}{\underset{i=1}{\sum }}\Upsilon_{i}$ and Γ = min{Γ_*i*_, *i* = 1, 2, ⋯, *M*}.

Integrating the Equation (67), one has


(68) $$\begin{array}{l} 0\leq V\leq e^{-\Gamma t}\left(V\left(0\right)-\frac{\Upsilon }{\Gamma }\right)+\frac{\Upsilon }{\Gamma } \end{array}$$

According to the definition of *V*, it can be included that *e*_*i,j*_, ${\tilde{\mu }}_{i,j}$ and ${\tilde{\eta }}_{i,m}$ all are bounded for *j* = 1, 2, ⋯, *m*, *i* = 1, 2, ⋯, *M*. Since μ_*i,j*_ and η_*i,m*_ are constant, then ${\tilde{\mu }}_{i,j}=\mu_{i,j}-{\hat{\mu }}_{i,j}$ and ${\tilde{\eta }}_{i,m}=\eta_{i,m}-{\hat{\eta }}_{i,m}$, it can be deduced that ${\hat{\mu }}_{i,j}$ and ${\hat{\eta }}_{i,m}$ are bounded. In addition, based on the definition of α_*i,j*_, it can be deduced that α_*i,j*_ is bounded, and existing up bound satisfies $|\alpha_{i,j}|\leq \alpha_{i,j}^{*}$. Based on the definition of *V*_*i,m*_, the following inequality holds:


(69) $$\begin{array}{l} \log\frac{\lambda_{i,j}^{2}}{\lambda_{i,j}^{2}-e_{i,j}^{2}}\leq e^{-\Gamma t}\left(V\left(0\right)-\frac{\Upsilon }{\Gamma }\right)+\frac{\Upsilon }{\Gamma } \end{array}$$

Thus, the solution *e*_*i,j*_ can be deduced as follows:


(70) $$\begin{array}{l} |e_{i,j}|\leq \lambda_{i,j}\sqrt{1-e^{-s}}<\lambda_{i,j} \end{array}$$

where $s=2\left(e^{-\Gamma t}\left(V\left(0\right)-\frac{\Upsilon }{\Gamma }\right)+\frac{\Upsilon }{\Gamma }\right)>0$

Then, combined with Equations (13), (18), one has


(71) $$\left|z_{i,j}\right|\leq \frac{\lambda_{i,j}}{g_{i}}=\{\begin{array}{ll} \left(1-Q^{-1}\right){\left(\frac{T-t}{T}\right)}^{2}\phi \left(t\right)\lambda_{i,j}+Q^{-1}\lambda_{i,j}, & \;0\leq t<T\\ Q^{-1}\lambda_{i,j}, & t\geq T \end{array}$$

Thus, all the signals are bounded, and error *z*_*i,j*_ converges to prescribed areas $\left\{z_{i,j}||z_{i,j}|\leq Q^{-1}\lambda_{i,j}\right\}$ within the prescribed time *T*.               □

*Proof of Theorem 1 (2)*. From Equation (71), it is obvious that $|z_{i,j}|\leq \lambda_{i,j}g_{i}^{-1}\left(t\right)$, according to the definition $\lambda_{i,j}g_{i}^{-1}\left(t\right)\leq Z_{i,j}^{v}\left(t\right)$, inequality $|z_{i,j}|\leq Z_{i,j}^{v}\left(t\right)$ is holds. Combined with $Z_{i,j}^{v}\left(t\right)\leq Z_{i,j}\left(t\right)$, it can be deduced that |*z*_*i,j*_| ≤ *Z*_*i,j*_(*t*) is always holds. Therefore, systems error *z*_*i,j*_ satisfies prescribed performance function *Z*_*i,j*_(*t*).

**Remark 6**. According to the aforementioned deduction and analysis, it is obvious that both the final convergence area $\left\{z_{i,j}||z_{i,j}|\leq Q^{-1}\lambda_{i,j}\right\}$ and convergence time *T* are prescribed. In addition system convergence progress satisfies prescribed performance function. From (70), it can be known that |*z*_*i,j*_| can become smaller, by increasing the design parameters γ_*i,j*_, β_*i,j*_, and ρ_*i,m*_ and decreasing design parameters δ_*i,j*_ and ξ_*i,m*_. However, both convergence time and convergence area are not affected by the adjustment of design parameters γ_*i,j*_, δ_*i,j*_, ξ_*i,m*_, β_*i,j*_, and ρ_*i,m*_.

In addition, according to Equation (71), it is obvious that $|z_{i,1}|\leq \frac{\lambda_{i,1}}{g_{i}}$. Applying **Lemma 3**, inequality $|y_{i}-y_{0}|\leq \frac{\lambda_{i,1}}{Q\varsigma_{\min}}$ is holds. As *y*_0_ satisfies *y*_0_ ≤ ȳ_0_, it can be obtained that $x_{i,1}\leq \frac{\lambda_{i,1}}{Q\varsigma_{\min}}+{ȳ}_{0}$. Define λ_*i*,1_ ≤ *Qς*_min_(*B*_*i*,1_−ȳ_*d*_), hence *x*_*i*,1_ ≤ *B*_*i*,1_ is holds.

Similarly, it is obvious that $|z_{i,2}|\leq \frac{\lambda_{i,2}}{g_{i}}$ holds from Equation (71). Due to *z*_*i*,2_ = *x*_*i*,2_ − α_*i*,1_, it can be obtained that $x_{i,2}\leq Q^{-1}\lambda_{i,2}+\alpha_{i,1}$. Consider the α_*i*,1_ satisfies $|\alpha_{i,1}|\leq \alpha_{i,1}^{*}$, and $x_{i,2}\leq Q^{-1}\lambda_{i,2}+\alpha_{i,1}^{*}$. Define $\lambda_{i,2}\leq Q\left(\alpha_{i,1}^{*}+B_{i,k}\right)$, hence *x*_*i*,2_ ≤ *B*_*i*,2_ is holds. Similarly, existing *x*_*i,j*_ ≤ *B*_*i,j*_ is holds for *j* = 3, ⋯, *m*

**Remark 7**. Most prescribed time control focuses on system convergence time but ignores the problem that, during rapid convergence progress, the system states may be out of safe ranges. To address the problem, by combining barrier Lyapunov function and transform function, achieving system quick convergence while ensuring state constraints.               □

*Proof of Theorem 1 (3)*. According to Equation (43), the derivative of Δ_*i*_(*t*) satisfies the following:


(72) $$\begin{array}{l} \frac{d}{dt}|\Delta_{i}\left(t\right)|\leq |{\dot{\varpi }}_{i}\left(t\right)|\leq {\overset{⌢}{\varpi }}_{i} \end{array}$$

Due to $\underset{t_{\omega }\to t_{\omega +1}}{\lim}$ Δ_*i*_(*t*) ≥ ϖ_*i*_(*t*)−*u*_*i*_(*t*) and Δ_*i*_(*t*_ω_) = 0, the following equation is obtained:


(73) $$\begin{array}{l} \underset{t_{\omega }\to t_{\omega +1}}{\lim}\frac{d}{dt}|\Delta_{i}\left(t\right)|\geq \frac{\zeta_{i}|{ū}_{i}\left(t\right)|+\sigma_{i}}{t_{\omega +1}-t_{\omega }}\geq \frac{\sigma_{i}}{t_{\omega +1}-t_{\omega }} \end{array}$$

Subsequently,


(74) $$\begin{array}{l} t_{\omega +1}-t_{\omega }\geq \frac{\sigma_{i}}{{\overset{⌢}{\varpi }}_{i}}>0 \end{array}$$

Thus, it can be concluded that interval time existing lower bound $\frac{\sigma_{i}}{{\overset{⌢}{\varpi }}_{i}}$ guarantees systems to avoid Zeno Behavior, and by increasing the design parameters σ_*i*_, the minimum interval time can be longer. Then, more communication resources can be saved by increasing design parameters ζ_*i*_.

The proof is completed.               □

The proposed theorem has been proved. By the proposed adaptive event-triggered consensus control method, it can be achieved that system convergence with prescribed performance while suffering from input dead zone and saturation.

**Remark 8**. Based on the prescribed performance function, the transform function and the barrier Lyapunov function are constructed and applied in each step. Therefore, the developed control method not only makes the system quickly converge in the prescribed area but also avoids states violating constrained. By introducing RBFNNs and designing adaptive laws, achieve unknown input dead-zone and saturation compensation in the prescribed time.

## 4. Simulation

**Figure 2:**
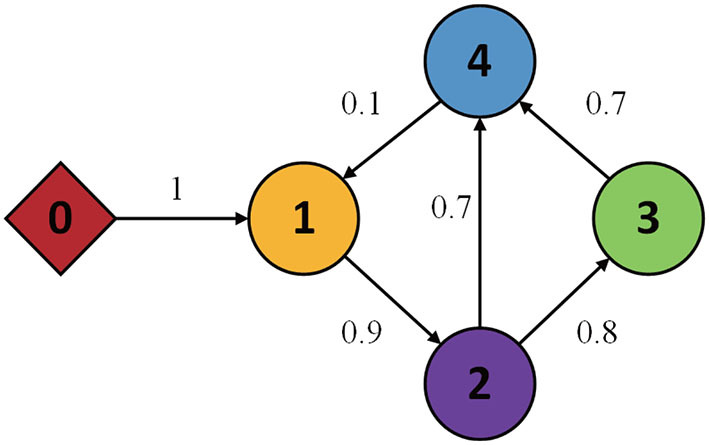
Topology of communication graph.

In this section, to prove the effectiveness of the control method developed in this study, some simulation experiments are presented. The MASs include four follower agents and one virtual leader is considered, and the topology of the communication graph is shown in Figure 2.

### 4.1. Example A

The model of *i*-*th*(i=1,2,3,4) follower is given as follows:


(75) $$\{\begin{array}{l} {\dot{x}}_{i,1}=x_{i,2}+f_{i,1}\left(X_{i,1}\right)\\ {\dot{x}}_{i,2}={\bar{u}}_{i}\left(u_{i}\right)+f_{i,2}\left(X_{i,2}\right)\\ y_{i}=x_{i,1} \end{array}$$

where unknown external disturbance expressed as *f*_*i*,1_(*X*_*i*,1_) = 0.2 sin(2*x*_*i*,1_) and *f*_*i*,2_(*X*_*i*,2_) = 0.2 sin(2*x*_*i*,1_*x*_*i*,2_). The dead-zone breakpoints are selected as *r*_*dl*_ = −0.8 and *r*_*dr*_ = 1, saturation breakpoints are selected as *r*_*sl*_ = −20 and *r*_*sr*_ = 25, and saturation values are *S*_*U*_ = 25 and *S*_*L*_ = −20. In addition, the unknown nonlinear functions are $D_{r}\left(u_{i}\right)=\frac{S_{U}}{r_{sr}-r_{dr}}\left(u_{i}-r_{dr}\right)$ and $D_{l}\left(u_{i}\right)=\frac{S_{L}}{r_{sl}-r_{dl}}\left(u_{i}-r_{dl}\right)$.

The prescribed time is selected as *T* = 1.2, and then, prescribed performance functions are selected as $Z_{i,1}=\left(5-0.05\right)e^{0.1t}+0.05$ and $Z_{i,2}=\left(10-0.1\right)e^{0.1t}+0.1$. Furthermore, transform function parameter is given as *Q* = 100. Define the values of state constraints as *x*_*i*,1_ < 2 and *x*_*i*,2_ < 5, then λ_*i*,1_ = 5 and λ_*i*,2_ = 10 are selected.

The developed adaptive event-triggered consensus control method is as follows:


(76) $$\begin{array}{l} \alpha_{i,1}=\frac{1}{\left(b_{i}+d_{i}\right)}\left(-\gamma_{i,1}z_{i,1}-z_{i,1}{\hat{\mu }}_{i,1}P_{i,1}\right) \end{array}$$

(77) $$\begin{array}{l} {\dot{\hat{\mu }}}_{i,1}=\frac{e_{i,1}^{2}}{\lambda_{i,1}^{2}-e_{i,1}^{2}}\beta_{i,1}P_{i,1}-\delta_{i,1}{\hat{\mu }}_{i,1} \end{array}$$

(78) $$\begin{array}{l} \alpha_{i,2}=-\gamma_{i,2}z_{i,2}-z_{i,2}{\hat{\mu }}_{i,2}P_{i,2}-\frac{\lambda_{i,2}^{2}-e_{i,2}^{2}}{g_{i}^{2}}z_{i,2} \end{array}$$

(79) $$\begin{array}{l} {\dot{\hat{\mu }}}_{i,2}=\frac{e_{i,2}^{2}}{\lambda_{i,2}^{2}-e_{i,2}^{2}}\beta_{i,2}P_{i,2}-\delta_{i,2}{\hat{\mu }}_{i,2} \end{array}$$

(80) $$\begin{array}{l} {\dot{\hat{\eta }}}_{i,2}=-\frac{e_{i,2}g_{i}\rho_{i,2}}{\lambda_{i,2}^{2}-e_{i,2}^{2}}\alpha_{i,2}-\xi_{i,2}{\hat{\eta }}_{i,2} \end{array}$$

(81) $$\{\begin{array}{c} \varpi_{i}\left(t\right)=-\left(1+\zeta_{i}\right)\left({\bar{\alpha }}_{i}\tanh\left(\frac{z_{i,2}{\bar{\alpha }}_{i}}{o_{i}}\right)+\frac{z_{i,2}}{2{\left(1-\zeta_{i}\right)}^{2}}\right)\\ u_{i}\left(t\right)=\varpi_{i}\left(t_{\omega }\right),\forall t\in \left[t_{\omega },t_{\omega +1}\right)\\ \Delta_{i}\left(t\right)=\varpi_{i}\left(t\right)-u_{i}\left(t\right)\\ t_{\omega +1}=\inf\left\{t\in R|\left|\Delta_{i}\left(t\right)\right|\geq \zeta_{i}\left|{\bar{u}}_{i}\left(t\right)\right|+\sigma_{i}\right\} \end{array}$$

The parameters of radial basis functions are selected as followers:


(82) $$\begin{array}{l} \Phi_{i}\left({\bar{X}}_{i}\right)=\exp\left(-\frac{{\left||{\bar{X}}_{i}-X_{i}^{*}|\right|}^{2}}{\pi_{i}^{2}}\right),i=1,2,\cdots ,5 \end{array}$$

where the inputs of the RBFNNs are ${\bar{X}}_{1,1}={\left[x_{1,1},x_{2,1},x_{2,2},\dot{y_{0}}\right]}^{T}$, ${\bar{X}}_{2,1}={\left[x_{2,1},x_{3,1},x_{3,2},\dot{y_{0}}\right]}^{T}$, ${\bar{X}}_{3,1}={\left[x_{3,1},x_{4,1},x_{4,2},\dot{y_{0}}\right]}^{T}$, ${\bar{X}}_{4,1}={\left[x_{4,1},x_{1,1},x_{1,2},\dot{y_{0}}\right]}^{T}$, ${\bar{X}}_{1,2}={\left[x_{1,1},x_{1,2},x_{2,1},x_{2,2},{\hat{\mu }}_{1,1}\right]}^{T}$, ${\bar{X}}_{2,2}={\left[x_{2,1},x_{2,2},x_{3,1},x_{3,2},{\hat{\mu }}_{2,1}\right]}^{T}$, ${\bar{X}}_{3,2}={\left[x_{3,1},x_{3,2},x_{4,1},x_{4,2},{\hat{\mu }}_{3,1}\right]}^{T}$, and ${\bar{X}}_{4,2}={\left[x_{1,1},x_{1,2},x_{2,1},x_{2,2},{\hat{\mu }}_{4,1}\right]}^{T}$. Then $\pi_{i}^{2}=2$. The parameters of event-triggered strategy are selected as ζ_*i*_ = 0.4, σ_*i*_ = 2.5, and *o*_*i*_ = 0.3, and remaining parameters and initial states are given in Table 1.

**Table 1:** System's initial states and controller parameters of Example A.

**Case**	***x*_*i*,1_**	***x*_*i*,2_**	**γ_*i*,1_**	**γ_*i*,2_**	**δ_*i,j*_**	**β_*i,j*_**	**ξ_*i*,2_**	**ρ_*i,m*_**
I	[0.2, 0.4, −0.2, −0.4]	[0, 0, 0, 0]	[20, 15, 14, 18]	[12, 10, 8, 4]	[2, 1.5]	[0.01, 0.05]	0.05	2
II	[0.3, 0.5, 0.8, 1]	[0.4, 0.8, 1.4, 1.2]	[20, 15, 14, 18]	[12, 10, 8, 4]	[2, 1.5]	[0.01, 0.05]	0.05	2
III	[0.3, 0.5, 0.8, 1]	[0.4, 0.8, 1.4, 1.2]	[25, 20, 15, 14]	[20, 15, 15, 10]	[1, 2]	[0.02, 0.01]	0.2	2.5

**Figure 3:**
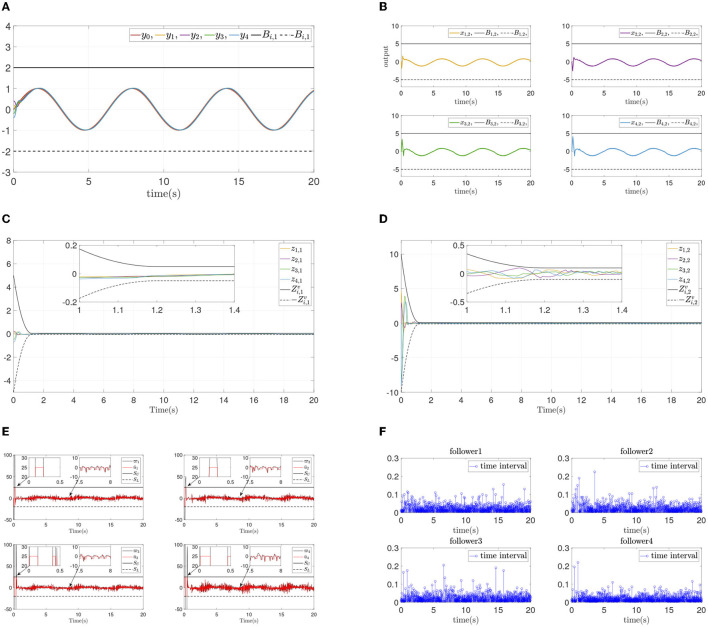
Simulations results of Example A case I. **(A)** Followers' outputs *y*_*i*_. **(B)** Followers' states *x*_*i*,2_. **(C)** Synchronization error *z*_*i*,1_. **(D)** Dynamics error *z*_*i*,2_. **(E)** systems input ū_*i*_ and event-triggered input ϖ_*i*_. **(F)** Time intervals.

The simulation results are shown in Figure 3. Figure 3A presents the follower output signal and leader output signal, and Figure 3B shows the states of each follower. From Figures 3A, B, it is obvious that followers track the leader quickiy, and state constraints are not violated at the same time. Figures 3C, D present the synchronization error and dynamics error of each follower. It can be seen that all errors converge in prescribed area within prescribed time (*T* = 1.2*s*), and convergence progress meet the prescribed performance. Figure 3E displays the systems input and event-triggered input, and it can be seen that the input is suffered from the input dead zone and saturation. And the triggered time intervals are presented in Figure 3F, it can be shown that the maximum interevent intervals from follower 1 to follower 4 are 0.15s, 0.22s, 0.20s, and 0.22s.

**Figure 4:**
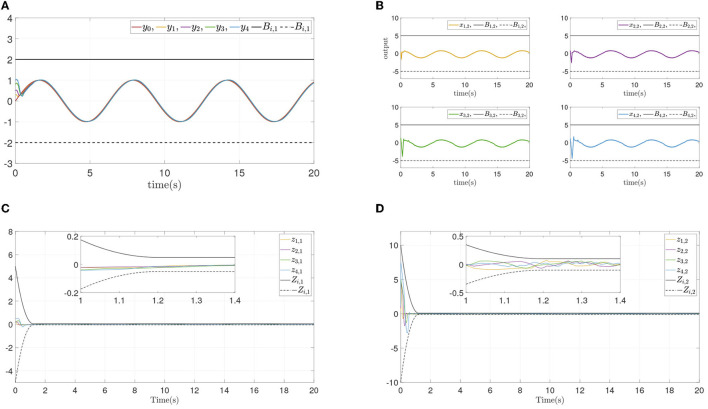
Simulation results of Example A case II. **(A)** Follower' outputs *y*_*i*_. **(B)** Followers' states *x*_*i*,2_. **(C)** Synchronization error *z*_*i*,1_. **(D)** Dynamics error *z*_*i*,2_.

**Figure 5:**
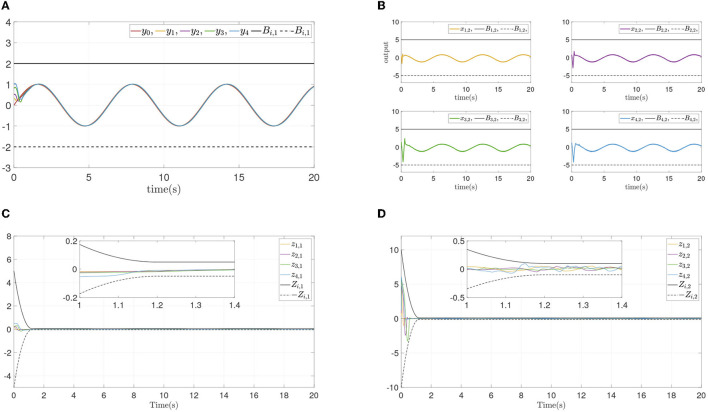
Simulation results of Example A case III. **(A)** Followers' outputs *y*_*i*_. **(B)** Followers' states *x*_*i*,2_. **(C)** Synchronization error *z*_*i*,1_. **(D)** Dynamics error *z*_*i*,2_.

To verify the convergence performance and time are not affected by systems initial states and control parameters, and ensure state constraints at the same time, two simulation experiments are done again for different design parameters and different initial states. The states and errors of two simulations are shown in Figures 4, 5. For different parameters or initial states, two similar results can be obtained: Each follower converge to the leader quickly while state constraints are not violated. All errors prescribed time convergence progress meet the prescribed performance. The simulation results verified the effectiveness of the developed control method.

### 4.2. Example B

To further verify the applicability of the constructed method, a class of constrained single-link robotic arm systems with input dead zone and saturation are adopted, and the models of *i*-*th*(i=1,2,3,4) arm are given as follows:


(83) $$\{\begin{array}{l} {\dot{x}}_{i,1}=x_{i,2}\\ {\dot{x}}_{i,2}=J_{i}^{-1}{\bar{u}}_{i}\left(u_{i}\right)-J_{i}^{-1}f_{i}\left(X\right)\\ y_{i}=x_{i,1} \end{array}$$

where *x*_*i*,1_ and *x*_*i*,2_, respectively, represent the *i*-*th* link angle and angular velocity. Then, *J*_*i*_ = 1 is the inertia moment. For *f*_*i*_(*X*) = (*B*_*i*_*x*_*i*,2_ + *G*_*i*_*L*_*i*_sin(*x*_*i*,1_)), *B*_*i*_ = 1 is the viscous friction coefficient, *G*_*i*_ = 9.8 and *l*_*i*_ = 0.1 represent the mass and length of the *i*-*th* link, $X={\left[x_{i,1},x_{i,2}\right]}^{T}$.

In Example B, a shorter prescribed time is selected as *T* = 1; then, prescribed performance functions are given as $Z_{i,1}=\left(3.75-0.05\right)e^{0.1t}+0.05$ and $Z_{i,2}=\left(7.5-0.1\right)e^{0.1t}+0.1$. Furthermore, transform function parameter is given as *Q* = 75. Define the same state constraints values as *x*_*i*,1_ < 2 and *x*_*i*,2_ < 5, then λ_*i*,1_ = 3.75 and λ_*i*,2_ = 7.5 are chosen. The parameters of the event-triggered strategy are the same as in Example A. The remaining parameters and initial states are given in Table 2.

**Table 2:** System's initial states and parameters of Example B.

**Case**	***x*_*i*,1_**	***x*_*i*,2_**	**γ_*i*,1_**	**γ_*i*,2_**	**δ_*i,j*_**	**β_*i,j*_**	**ξ_*i*,2_**	**ρ_*i,m*_**
I	[0.2, 0.3, −0.2, −0.3]	[0.1, 0.2, 0.3, 0.4]	[24, 28, 14, 20]	[16, 18, 16, 14]	[3, 2]	[0.01, 0.01]	0.1	2.5
II	[0.2, 0.4, 0.2, 0.6]	[0.4, 0.3, 0.2, 0.1]	[24, 28, 14, 20]	[16, 18, 16, 14]	[3, 2]	[0.01, 0.01]	0.1	2.5
III	[0.2, 0.4, 0.2, 0.6]	[0.4, 0.3, 0.2, 0.1]	[30, 20, 15, 18]	[25, 15, 20, 12]	[5, 4]	[0.03, 0.2]	0.2	3

**Figure 6:**
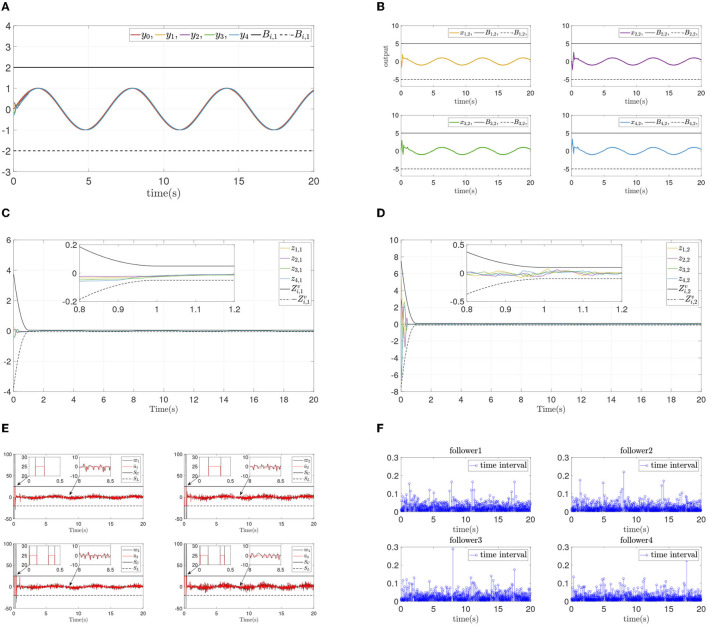
Simulation results of Example B case I. **(A)** Followers' outputs *y*_*i*_. **(B)** Followers' states *x*_*i*,2_. **(C)** Synchronization error *z*_*i*,1_. **(D)** Dynamics error *z*_*i*,2_. **(E)** systems input ū_*i*_ and event-triggered input ϖ_*i*_. **(F)** Time intervals.

**Figure 7:**
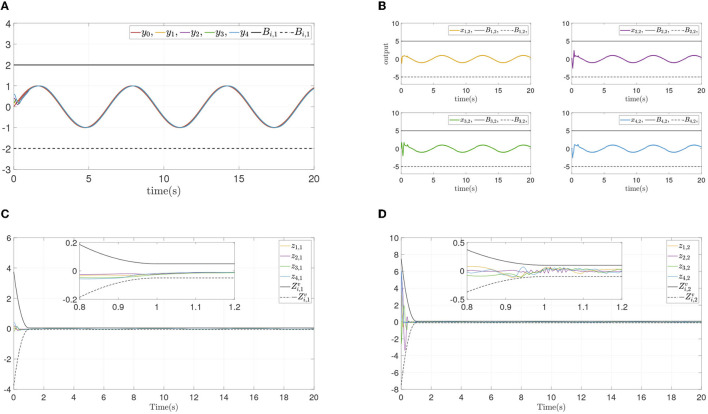
Simulation results of Example B case II. **(A)** Followers' outputs *y*_*i*_. **(B)** Followers' states *x*_*i*,2_. **(C)** Synchronization error *z*_*i*,1_. **(D)** Dynamics error *z*_*i*,2_.

**Figure 8:**
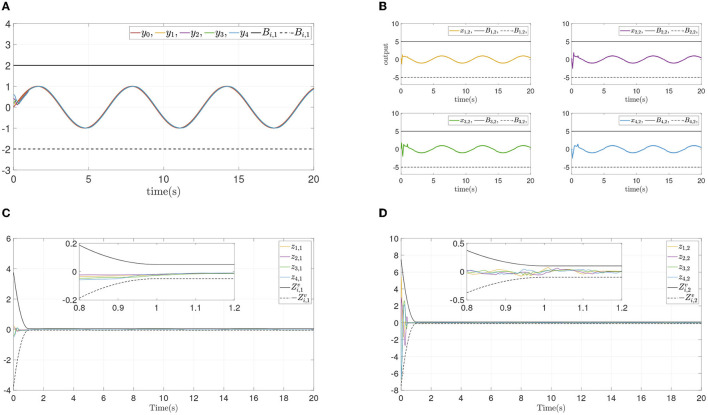
Simulation results of Example B case III. **(A)** Followers' outputs *y*_*i*_. **(B)** Followers' states *x*_*i*,2_. **(C)** Synchronization error *z*_*i*,1_. **(D)** Dynamics error *z*_*i*,2_.

From the simulation result in Figure 6, it can be seen that similar control performance in Example A is shown. Considering different design parameters and different initial states in Table 2, two simulation experiments are done ulteriorly. The system states and error convergence progress are displayed in Figures 7, 8. According to the result from Figures 6–8, it is obvious that system quickly converges in the prescribed area with prescribed performance while affected by input dead zone and saturation, and state constraints are guaranteed. Therefore, the effectiveness of the proposed control method is confirmed.

## 5. Conclusion

This study has discussed the prescribed performance consensus problem of constrained multiagent systems with input dead zone and saturation. An adaptive event-triggered consensus control method is developed to address the problem. To compensate for the impact caused by unknown input dead zone and saturation, adaptive laws and RBFNNs are adopted to deal with them. Based on constructed transform function and barrier Lyapunov function, the control method is designed by backstepping technology, which guarantees the system convergence performance and prevents constraint violation. Under the proposed control method, all followers achieve prescribed time and preset precision synchronization, irrespective of the presence of limited bandwidth, input dead zone, and saturation. Some simulations show the feasibility of the proposed control method. In future studies, we tend to discuss the consensus problem when the parameters of input dead zone and saturation are unknown and time-varying. Moreover, how to compensate for actuator failures and the input time delay is also an interesting topic that merits research.

## Data availability statement

The original contributions presented in the study are included in the article/supplementary material, further inquiries can be directed to the corresponding author.

## Author contributions

XY and JL: conceptualization, writing—original draft preparation, data curation, writing—review and editing, and funding acquisition. XY, JL, and KC: methodology. KC: validation and supervision. YZ and ZH: formal analysis and project administration. All authors have read and agreed to the published version of the manuscript.
